# National Trends in Cessation Counseling, Prescription Medication Use, and Associated Costs Among US Adult Cigarette Smokers

**DOI:** 10.1001/jamanetworkopen.2019.4585

**Published:** 2019-05-24

**Authors:** Martin Tibuakuu, Victor Okunrintemi, Ermias Jirru, Justin B. Echouffo Tcheugui, Olusola A. Orimoloye, Puja K. Mehta, Andrew P. DeFilippis, Michael J. Blaha, Erin D. Michos

**Affiliations:** 1 St Luke’s Hospital, Department of Medicine, Chesterfield, Missouri; 2 Ciccarone Center for the Prevention of Cardiovascular Disease, Johns Hopkins University School of Medicine, Baltimore, Maryland; 3 Department of Internal Medicine, East Carolina University, Greenville, North Carolina; 4 Department of Medicine, New York Presbyterian-Weill Cornell Medical College, New York; 5 Department of Medicine, Johns Hopkins University, Baltimore, Maryland; 6 Emory Clinical Cardiovascular Research Institute, Atlanta, Georgia; 7 Division of Cardiology, University of Louisville, Louisville, Kentucky

## Abstract

**Importance:**

Cigarette smoking is the leading cause of preventable disease and death in the United States. When used separately or in combination, smoking cessation counseling and cessation medications have been associated with increased cessation rates.

**Objectives:**

To present trends in self-reported receipt of physician advice to quit smoking and in use of prescription smoking cessation medication along with their associated expenditures among a nationally representative sample of active adult smokers in the United States.

**Design, Setting, and Participants:**

This repeated cross-sectional study of US adults aged 18 years or older was conducted from July 5, 2018, through August 15, 2018. Data were collected between January 1, 2006, and December 31, 2015, from the Medical Expenditure Panel Survey, an annual US survey of individuals and families, health care personnel, and employers. Participants (n = 29 106) were noninstitutionalized civilians who were randomly drawn from the respondents of the previous year’s National Health Interview Survey. Multivariable logistic regression models were used to examine the associations between sociodemographic factors and receipt of physician cessation advice and use of cessation prescription medication. A 2-part econometric model was used to assess health care expenditures.

**Main Outcomes and Measures:**

Trends in self-reported receipt of physician advice to quit and uptake of prescription smoking cessation medications with associated total and out-of-pocket expenditures.

**Results:**

The study sample consisted of 29 106 participants, with a mean (SD) age of 57 (10) years and a composition of 13 670 women (47.0%). The results were weighted to provide estimates for 31.2 million active adult cigarette smokers. The proportion of smokers who reported receiving physician advice to quit increased from 60.2% (95% CI, 58.5%-62.0%) in 2006 to 2007 to 64.9% (95% CI, 62.8%-66.9%) in 2014 to 2015, with a *P* for trend = .001. The odds of receiving physician cessation advice was statistically significantly higher in women (odds ratio [OR], 1.50; 95% CI, 1.39-1.59) and lower among uninsured participants (OR, 0.58; 95% CI, 0.52-0.65). Overall, prescription smoking cessation medication use decreased with a corresponding reduction in total expenditures from $146 million (out-of-pocket cost, $46 million) in 2006 to 2007 to $73 million (out-of-pocket cost, $9 million) in 2014 to 2015. Male (odds ratio [OR], 0.78; 95% CI, 0.66-0.91), uninsured (OR, 0.58; 95% CI, 0.41-0.83), and racial/ethnic minority (African American: OR, 0.51 [95% CI, 0.38-0.69]; Asian: OR, 0.31 [95% CI, 0.10-0.93]; Hispanic: OR, 0.53 [95% CI, 0.36-0.78]) participants were less likely to use prescription smoking cessation medications.

**Conclusions and Relevance:**

The lower rates of delivery of physician advice to quit smoking and the lower uptake of known prescription smoking cessation medications among men, younger adults, uninsured individuals, racial/ethnic minority groups, and those without smoking-associated comorbidities may be associated with the higher smoking rates among these subgroups despite an all-time low prevalence of smoking in the United States; this finding calls for a more targeted implementation of smoking cessation guidelines.

## Key Points

**Question:**

What are the 10-year trends in the delivery of cigarette smoking cessation services for adult smokers in the United States?

**Findings:**

In this cross-sectional study of 29 106 US adult smokers from 2006 to 2015, the proportion who reported receiving physician advice to quit smoking increased and the use of prescription cessation medication decreased with a corresponding reduction in expenditures. Lower rates of cessation services were observed in certain populations, including men, younger adults, uninsured individuals, racial/ethnic minority groups, and those without smoking-related comorbidities.

**Meaning:**

The disparities in the delivery of known smoking cessation services among certain subgroups may be associated with higher smoking rates despite an all-time low prevalence of smoking, suggesting the call for a more targeted implementation of smoking cessation guidelines.

## Teaser

This cross-sectional study of multiyear Medical Expenditure Panel Survey data evaluates the association between socioeconomic factors, availability of cessation information or services, and cigarette smoking rates in adults in the United States.

## Introduction

Cigarette smoking remains the leading cause of preventable disease and death in the United States despite the considerable decrease in smoking prevalence since the release more than 50 years ago of the first US Surgeon General’s report on the health implications of cigarette smoking.^1,2,3,4,5,6^ An estimated 480 0000 US residents die of smoking-related illnesses each year.^1,2^ Overall, smoking costs the nation at least $130 billion in medical care and more than $150 billion in lost productivity annually.^1^ Consequently, federally sponsored programs, like Medicare, and private health plans have an enormous economic burden.^1,7^ An estimated 60% of the US health care spending attributable to cigarette smoking is paid by public programs.^7^

Smoking cessation substantially reduces smoking-related morbidity and mortality.^1,2,3,8,9,10^ When used separately or in combination, smoking cessation counseling and cessation pharmacotherapies are associated with higher cessation rates.^9^ Even brief cessation advice or counseling by health care professionals has been associated with effectiveness.^9^ Thus, clinical guidelines recommend that smoking cessation counseling be addressed at every clinical visit.^9^

In the United States, 7 US Food and Drug Administration–approved smoking cessation pharmacotherapies are available, including 5 forms of nicotine replacement therapy (NRT) as well as bupropion hydrochloride and varenicline tartrate. The NRTs are available over the counter (OTC), but the 2 non-NRT medications are prescription only. Although each of these agents is more efficacious than placebo in smoking cessation,^11^ clinical trials and meta-analyses have demonstrated superior efficacy of prescription non-NRT, especially varenicline, as compared with OTC NRT.^12,13^ Thus, the 2018 American College of Cardiology Expert Consensus Decision Pathway on Tobacco Cessation Treatment guidelines recommend prescription varenicline as the first-line pharmacotherapeutic agent for smoking cessation.^14^ Nonetheless, data are lacking on trends in the uptake of prescription smoking cessation therapies, and their associated costs, among the general population of US adult smokers.

The objective of this study was to present the 10-year trends in physician advice to quit smoking and prescription smoking cessation medication use along with their associated total and out-of-pocket expenditures in a nationally representative sample of adult active smokers in the United States.

## Methods

This cross-sectional study was considered exempt from institutional review board approval by the US Department of Health and Human Services because the Medical Expenditure Panel Survey (MEPS) data used were publicly available and deidentified. All participants in the data source, the MEPS, provided written informed consent to be interviewed and for their health data to be obtained from their physicians and pharmacies. This study followed the Strengthening the Reporting of Observational Studies in Epidemiology (STROBE) reporting guideline for cross-sectional studies.

### Study Population and Design

Sponsored by the Agency for Healthcare Research and Quality, the MEPS is an annual cross-sectional national survey of individuals and families, health care personnel, and employers that provides information on sociodemographic factors, medical conditions, health care resource use, and health care expenditures.^15,16^ Participants in each annual household component of the MEPS are randomly drawn from the previous year’s National Health Interview Survey and consist of noninstitutionalized US civilians.

After the sample draw, participants are interviewed over the telephone, and their data (including sociodemographic characteristics, patient experience, medical conditions, prescription medications, health resource use, associated costs, and payment sources) are reported in different data files.^15,16^ Additional information on health care use and cost are collected from physicians, hospitals, and pharmacies. To ensure national representativeness, person-weight and variance-estimation stratum are assigned to each respondent to account for survey nonresponse and the characteristics of the national population of the survey year.^15,16^ Additional data on health care use and cost are obtained from physicians, hospitals, and pharmacies.^15,16^

Analyses for the present study, conducted from July 5, 2018, through August 15, 2018, were based on 10 years of the MEPS data. These data were collected from participants aged 18 years or older from January 1, 2006, to December 31, 2015.

### Cessation Counseling and Pharmacotherapy Use and Expenditure

Information on cigarette smoking and cessation counseling was collected during the MEPS household interview.^17^ Participants were asked the following questions: Do you currently smoke? If yes, has your doctor advised you to quit smoking? All of the variables referred to events experienced in the past 12 months.

Participants reported the names of any smoking cessation prescription medications they or their family members purchased or acquired during the past 12 months. The names of these medications and other information reported were entered verbatim by the MEPS interviewers.

With the written permission of the MEPS participants who responded to the smoking and cessation questions, the interviewers contacted pharmacies to obtain further details on medication name, date filled, national drug code, strength, and quantity; exact dollar amount paid; and source of payment, including out of pocket and specific insurance coverage. No information was collected for OTC medications. The 2006 through 2015 MEPS did not ascertain electronic cigarette use.

### Other Variables

The sociodemographic factors considered in this study were sex, age category (18-39, 40-64, 65-74, or ≥75), self-reported race/ethnicity (non-Hispanic white, African American, Hispanic, Asian, or other), family income level (poor or very low income: <125% of the federal poverty level [FPL]; low income: 125% to <200% FPL; middle income: 200% to <400% FPL; or high income: ≥400% FPL), educational level (<high school, high school or GED [General Education Development] equivalent, or ≥ college), insurance status (public, private, or uninsured), and census region (Northeast, Midwest, South, or West). We also examined the presence of a diagnosis of 2 smoking-related comorbid conditions: (1) atherosclerotic cardiovascular disease (ASCVD) (coronary artery disease, stroke, and/or peripheral artery disease), and (2) chronic obstructive pulmonary disease (COPD). Both conditions were ascertained by self-report and/or use of the *International Classification of Diseases, Ninth Revision, Clinical Modification* codes (eTable 1 in the Supplement).

### Statistical Analysis

We pooled the MEPS data into 2-year survey cycles (2006-2007, 2008-2009, 2010-2011, 2012-2013, and 2014-2015) for ease of analysis and reporting. Person-level weight was adjusted accordingly to reflect the mean annual population size, medication use, and expenditures over the 2 years in each cycle. All analyses were conducted using Stata, version 15 (StataCorp LLC), and accounted for the complex MEPS sampling and survey design. Using Stata’s *svy* prefix command, we weighted the results to provide nationally representative estimates and their 95% CIs for the proportions of civilian noninstitutionalized adults in the United States by their sociodemographic and comorbid factors.

First, frequency distributions and weighted proportions of the sample characteristics were summarized across the five 2-year cycles. Comparisons were made using analysis of variance for means and the Kruskal-Wallis test for proportions. Using descriptive statistics, we estimated the weighted proportions of physician advice to quit smoking and cessation pharmacotherapy use per cycle. These proportions were plotted for the whole cohort and by subgroups.

Second, progressively adjusted multivariable logistic regression models were used to determine the associations of sociodemographic factors and the presence of smoking-related comorbidities with physician advice to quit smoking and with cessation pharmacotherapy use. Results were reported as odds ratios (ORs) and 95% CIs. Model 1 was adjusted for age, sex, and race/ethnicity. Model 2 was adjusted for all variables listed in Table 1. Reported CIs and tests used design-based estimates of variance.

**Table 1.** Characteristics of US Smokers Aged 18 Years or Older, 2006 to 2015 MEPS

Variable	No. (%)					*P* Value^a^
	2006-2007 MEPS Cycle	2008-2009 MEPS Cycle	2010-2011 MEPS Cycle	2012-2013 MEPS Cycle	2014-2015 MEPS Cycle	
No. of adults	5878	6179	5847	6172	5030	
Weighted sample, million	6.4	6.7	6.3	6.2	5.6	
Age, mean (SD), y	43.6 (14.9)	42.8 (14.9)	43.5 (15.1)	43.5 (15.0)	44.2 (15.2)	<.001
Age category, y						
18-39	3202 (40.7)	3520 (43.7)	3319 (42.9)	3499 (43.4)	2793 (43.1)	<.001
40-64	3973 (50.5)	3914 (48.6)	3682 (47.6)	3846 (47.7)	3037 (46.8)	
65-74	492 (6.2)	456 (5.7)	531 (6.9)	564 (7.0)	503 (7.8)	
≥75	207 (2.6)	170 (2.1)	198 (2.6)	160 (2.0)	153 (2.4)	
Female sex	3702 (47.0)	3733 (46.3)	3575 (46.2)	3680 (45.6)	2948 (45.5)	.32
Race/ethnicity						
Non-Hispanic white	4794 (63.0)	4425 (56.7)	4231 (56.6)	3945 (50.5)	3122 (50.2)	<.001
African American	1420 (18.7)	1755 (22.5)	1772 (23.7)	2110 (27.0)	1777 (28.5)	
Asian	170 (2.2)	260 (3.3)	294 (3.9)	323 (4.1)	262 (4.2)	
Hispanic	1222 (16.1)	1361 (17.4)	1177 (15.7)	1434 (18.4)	1064 (17.1)	
Health insurance status						
Private	3548 (48.2)	3473 (46.1)	3132 (42.0)	3266 (40.5)	2695 (41.6)	<.001
Uninsured	2029 (27.6)	2212 (29.4)	2148 (28.8)	2200 (27.3)	1255 (19.4)	
Medicaid	1063 (14.4)	1190 (15.8)	1464 (19.6)	1887 (23.4)	1885 (29.1)	
Medicare	720 (9.8)	656 (8.7)	713 (9.6)	709 (8.8)	644 (9.9)	
Family income level^b^						
High	1741 (22.1)	1614 (20.0)	1322 (17.1)	1391 (17.2)	1084 (16.7)	<.001
Middle	2355 (29.9)	2380 (29.5)	2291 (29.6)	2261 (28.0)	1786 (27.5)	
Low	1368 (17.4)	1484 (18.4)	1382 (17.9)	1469 (18.2)	1171 (18.1)	
Very low	2410 (30.6)	2582 (32.0)	2735 (35.4)	2948 (36.5)	2445 (37.7)	
Census region						
Northeast	1072 (13.6)	1184 (14.7)	1127 (14.6)	1300 (16.1)	1026 (15.8)	<.001
Midwest	1974 (25.1)	1989 (24.7)	1969 (25.5)	1857 (23.0)	1581 (24.4)	
South	3234 (41.1)	3304 (41.0)	3260 (42.2)	3415 (42.3)	2687 (41.4)	
West	1594 (20.2)	1583 (19.6)	1374 (17.8)	1497 (18.6)	1192 (18.4)	
Comorbid condition						
ASCVD	696 (8.8)	895 (11.1)	847 (11.0)	894 (11.1)	776 (12.0)	<.001
COPD	531 (8.1)	755 (11.3)	712 (11.2)	694 (10.4)	611 (11.4)	

Abbreviations: ASCVD, atherosclerotic cardiovascular disease (coronary artery disease, stroke, and/or peripheral artery disease); COPD, chronic obstructive pulmonary disease; MEPS, Medical Expenditure Panel Survey.

^a^ *P* values were computed using analysis of variance for means and the Kruskal-Wallis test for proportions.

^b^ The Other Variables section in Methods indicates the meaning of the levels.

A 2-part econometric model was used to study health care expenditures, adjusted to 2015 US dollars using the gross domestic product index.^18^ *P* values were 2-sided and considered statistically significant if *P* < .05.

## Results

Of the 340 292 individuals sampled in the MEPS from 2006 to 2015, a total of 29 106 (8.5%) were current smokers. The smokers included 13 670 women (47.0%), with a mean (SD) age of 57 (10) years. Results were weighted to represent approximately 31.2 million noninstitutionalized adult active smokers in the United States (eFigure 1 in the Supplement). A description of the characteristics of the study population by survey cycle is shown in Table 1.

**Figure 1.**
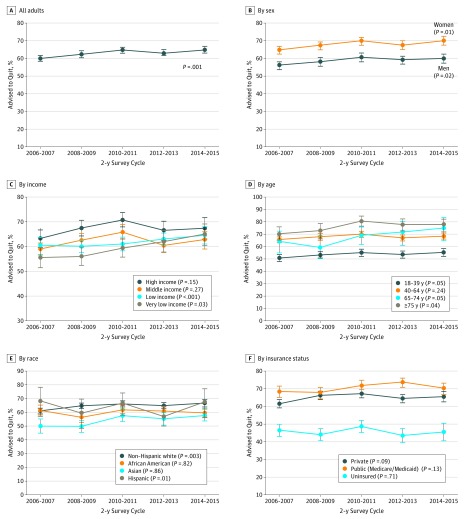
Trends in Weighted Proportions of US Adult Smokers Reporting Physician Advice to Quit Smoking, 2006-2015 Error bars represent 95% CIs. Data are from the Medical Expenditure Panel Survey.

The 10-year trends of the proportion of participants who self-reported receiving their physician’s advice to quit smoking among all active smokers and by different subgroups are shown in Figure 1 and eFigure 2 and eTable 2 in the Supplement. The proportion of adult smokers who received physician advice to quit at any clinical visit within the past year increased from 60.2% (95% CI, 58.5%-62.0%) in 2006 to 2007 to 64.9% (95% CI, 62.8%-66.9%) in 2014 to 2015 (*P* for trend = .001; eTable 2 in the Supplement). Smoking cessation counseling increased for both men and women from 2006 to 2015 but remained consistently lower in men than in women across all 5 cycles (56.1% [95% CI, 53.8%-58.3%] vs 65.0% [95% CI, 62.8%-67.2%] in 2006-2007; 60.0% [95% CI, 57.3%-62.6%] vs 70.3% [95% CI, 67.6%-72.8%] in 2014-2015).

Similarly, receipt of advice to quit remained statistically significantly lower among uninsured adult smokers compared with smokers with either public or private health insurance (46.3% [95% CI, 42.7%-49.8%] for uninsured vs 68.3% [95% CI, 65.1%-71.4%] for public insurance in 2006-2007; 45.4% [95% CI, 40.3%-50.7%] for uninsured vs 70.0% [95% CI, 66.9%-72.9%] for public insurance in 2014-2015). Disparities in smoking cessation counseling were also noted for lower-income, younger, and Hispanic individuals. Among individuals with ASCVD over this 10-year period, the rate of cessation counseling plateaued and never increased above 82.3% (95% CI, 77.8%-86.1%), and for those with COPD, the rate was 85.5% (95% CI, 81.3%-88.8%).

Table 2 shows the odds of receiving physician advice to quit smoking in the past year among adult smokers. In multivariable-adjusted analysis (model 2), the odds of reporting the receipt of physician advice to quit were higher among adults aged 40 to 64 years (OR, 1.55; 95% CI, 1.42-1.69) and those aged 65 to 74 years (OR, 1.45; 95% CI, 1.15-1.83) compared with adults aged 18 to 39 years. Women (compared with men) were more likely to report receiving physician advice to quit (OR, 1.50; 95% CI, 1.39-1.59), as were Asian individuals (OR, 1.39; 95% CI, 1.05-1.83), compared with non-Hispanic white individuals.

**Table 2.** Odds Ratios of Receiving Smoking Cessation Services Among US Adult Smokers, 2006 to 2015 Medical Expenditure Panel Survey

Variable	Odds Ratio (95% CI)					
	Physician Advice to Quit Smoking			Use of Prescription Smoking Cessation Medication		
	Univariate	Model 1^a^	Model 2^b^	Univariate	Model 1^a^	Model 2^b^
MEPS cycle						
2006-2007	1 [Reference]	1 [Reference]	1 [Reference]	1 [Reference]	1 [Reference]	1 [Reference]
2008-2009	1.10 (0.99-1.21)	1.09 (0.99-1.21)	1.06 (0.94-1.20)	0.87 (0.70-1.08)	0.87 (0.70-1.09)	0.81 (0.64-1.02)
2010-2011	1.22 (1.10-1.36)	1.23 (1.10-1.37)	1.17 (1.00-1.36)	0.81 (0.64-1.02)	0.80 (0.63-1.02)	0.75 (0.54-1.04)
2012-2013	1.13 (1.02-1.25)	1.13 (1.01-1.25)	1.04 (0.89-1.21)	0.76 (0.61-0.95)	0.76 (0.67– 0.96)	0.68 (0.49-0.93)
2014-2015	1.22 (109-1.37)	1.20 (1.07-1.35)	1.09 (0.94-1.26)	0.91 (0.72-1.16)	0.90 (0.70-1.16)	0.70 (0.51-0.97)
Age category, y						
18-39	1 [Reference]	1 [Reference]	1 [Reference]	1 [Reference]	1 [Reference]	1 [Reference]
40-64	1.83 (1.71-1.95)	1.81 (1.70–1.93)	1.55 (1.42-1.69)	1.54 (1.27-1.86)	1.53 (1.27–1.86)	1.42 (1.15-1.76)
65-74	2.82 (2.46-3.23)	2.84 (2.46–3.28)	1.45 (1.15-1.83)	1.24 (0.87-1.76)	1.25 (0.88–1.78)	0.90 (0.54-1.97)
≥75	1.85 (1.53-2.24)	1.83 (1.51–2.22)	1.00 (0.74-1.35)	0.56 (0.26-1.22)	0.52 (0.23–1.17)	0.50 (0.16-1.55)
Sex						
Male	1 [Reference]	1 [Reference]	1 [Reference]	1 [Reference]	1 [Reference]	1 [Reference]
Female	1.49 (1.40-1.60)	1.52 (1.41–1.61)	1.50 (1.39-1.59)	1.28 (1.10-1.52)	1.28 (1.10–1.49)	1.28 (1.10-1.52)
Race/ethnicity						
Non-Hispanic white	1 [Reference]	1 [Reference]	1 [Reference]	1 [Reference]	1 [Reference]	1 [Reference]
African American	0.82 (0.75-0.90)	0.84 (0.77–0.91)	1.01 (0.91-1.12)	0.49 (0.39-0.62)	0.49 (0.39-0.61)	0.51 (0.38-0.69)
Asian	0.97 (0.79-1.20)	1.11 (0.90–1.37)	1.39 (1.05-1.83)	0.23 (0.09-0.58)	0.24 (0.10-0.62)	0.31 (0.10-0.93)
Hispanic	0.65 (0.58-0.71)	0.73 (0.66–0.81)	0.84 (0.74-0.95)	0.53 (0.39-0.72)	0.55 (0.41-0.75)	0.53 (0.36-0.78)
Insurance status						
Private	1 [Reference]	1 [Reference]	1 [Reference]	1 [Reference]	1 [Reference]	1 [Reference]
Public	1.31 (1.21-1.42)	1.23 (1.12-1.35)	1.11 (0.97-1.27)	0.92 (0.78-1.08)	0.49 (0.39-0.61)	1.18 (0.91-1.54)
Uninsured	0.45 (0.42-0.50)	0.50 (0.45-0.54)	0.58 (0.52-0.65)	0.44 (0.33-0.60)	0.24 (0.10-0.62)	0.58 (0.41-0.83)
Level of income						
High	1 [Reference]	1 [Reference]	1 [Reference]	1 [Reference]	1 [Reference]	1 [Reference]
Middle	0.81 (0.73-0.89)	0.85 (0.76-0.94)	0.89 (0.78-1.02)	0.91 (0.75-1.10)	0.95 (0.79-1.16)	1.00 (0.80-1.24)
Low	0.73 (0.66-0.80)	0.75 (0.68-0.84)	0.79 (0.68-0.97)	0.73 (0.58-0.92)	0.79 (0.62-1.00)	0.82 (0.60-1.13)
Very low	0.80 (0.73-0.87)	0.86 (0.78-0.95)	0.84 (0.73-0.97)	0.82 (0.67-0.99)	0.90 (0.73-1.11)	0.88 (0.65-1.20)
Educational status						
<High school	1 [Reference]	1 [Reference]	1 [Reference]	1 [Reference]	1 [Reference]	1 [Reference]
High school/GED equivalent	1.05 (0.97-1.14)	1.00 (0.92-1.09)	0.96 (0.87-1.06)	1.15 (0.92-1.43)	0.95 (0.79-1.16)	1.04 (0.81-1.33)
≥College	1.22 (1.10-1.36)	1.13 (1.01-1.26)	1.05 (0.92-1.20)	1.40 (1.07-1.83)	0.79 (0.62-1.00)	1.34 (1.01-1.79)
Comorbid condition						
No ASCVD	1 [Reference]	1 [Reference]	1 [Reference]	1 [Reference]	1 [Reference]	1 [Reference]
ASCVD	2.78 (2.44-3.13)	2.38 (2.08-2.70)	2.08 (1.79-2.50)	1.45 (1.19-1.75)	1.44 (1.18-1.79)	1.35 (1.05-1.75)
No COPD	1 [Reference]	1 [Reference]	1 [Reference]	1 [Reference]	1 [Reference]	1 [Reference]
COPD	2.86 (2.56-3.33)	2.50 (2.19-2.88)	2.38 (2.00-2.78)	1.88 (1.54-2.38)	1.69 (1.39-2.08)	1.61 (1.27-2.04)
Census region						
Northeast	1 [Reference]	1 [Reference]	1 [Reference]	1 [Reference]	1 [Reference]	1 [Reference]
Midwest	0.71 (10.63-0.81)	0.69 (0.61-0.79)	0.74 (0.63-0.87)	1.08 (0.83-1.42)	1.08 (0.82-1.43)	1.29 (0.90-1.83)
South	0.62 (0.55-0.69)	0.59 (0.52-0.67)	0.67 (0.58-0.78)	0.81 (0.62-1.05)	0.83 (0.63-1.08)	0.88 (0.63-1.25)
West	0.64 (0.55-0.74)	0.64 (0.56-0.75)	0.68 (0.57-0.81)	0.93 (0.70-1.25)	1.00 (0.74-1.35)	1.00 (0.68-1.48)

Abbreviations: ASCVD, atherosclerotic cardiovascular disease (coronary artery disease, stroke, and/or peripheral artery disease); COPD, chronic obstructive pulmonary disease; GED, General Education Development; MEPS, Medical Expenditure Panel Survey.

^a^ Model 1: adjusted for age, sex, and race/ethnicity along with the univariate factor.

^b^ Model 2: all variables in the Table were included in this model.

In contrast, odds of reporting the receipt of physician advice to quit smoking were lower for Hispanic individuals compared with non-Hispanic white individuals (OR, 0.84; 95% CI, 0.74-0.95); uninsured individuals compared with those privately insured (OR, 0.58; 95% CI, 0.52-0.65); and for low-income individuals (OR, 0.79; 95% CI, 0.68-0.97) or very-low-income (OR, 0.84; 95% CI, 0.73-0.97) individuals compared with high-income individuals. Individuals with ASCVD (OR, 2.08; 95% CI, 1.79-2.50) and COPD (OR, 2.38; 95% CI, 2.00-2.78), compared with those without these conditions, were more likely to report being advised to quit. Compared with smokers in the Northeast region, those in the Midwest (OR, 0.74; 95% CI, 0.63-0.87), South (OR, 0.67; 95% CI, 0.58-0.78), and West (OR, 0.68; 95% CI, 0.57-0.81) were less likely to have received physician advice to quit.

**Figure 2.**
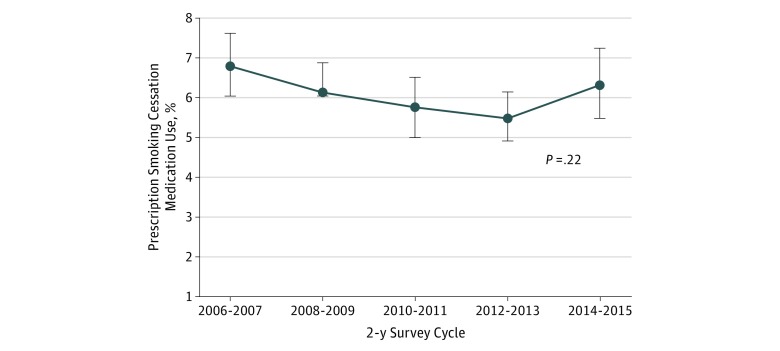
Trends in Weighted Proportions of US Adult Smokers Using Prescription Smoking Cessation Medications, 2006-2015 Error bars represent 95% CIs. Data from the Medical Expenditure Panel Survey.

The 10-year trends of the proportions of individuals who used prescription smoking cessation medications among all current adult smokers and by subgroups are shown in Figure 2 and eTable 3 in the Supplement. The proportion of adult smokers using prescription smoking cessation medications decreased from 6.0% (95% CI, 5.2%-6.9%) in 2006 to 2007 to 4.6% (95% CI, 4.0%-5.3%) in 2012 to 2013. This proportion increased to 5.5% (95% CI, 4.6%-6.5%) in 2014 to 2015 (eTable 3 in the Supplement).

Table 2 also shows the odds for having taken prescription smoking cessation medication in the past year among active adult smokers. Compared with the odds in 2006 to 2007, the odds of using prescription smoking cessation medication were statistically significantly lower in 2012 to 2013 (OR, 0.68; 95% CI, 0.49-0.93) and 2014 to 2015 (OR, 0.70; 95% CI, 0.51-0.97). Women (OR, 1.28; 95% CI, 1.10-1.52) who were active smokers were more likely to be taking prescription smoking cessation medication compared with men. Racial/ethnic minority groups, including African American (OR, 0.51; 95% CI, 0.38-0.69), Asian (OR, 0.31; 95% CI, 0.10-0.93), and Hispanic (OR, 0.53; 95% CI, 0.36-0.78]), were less likely to use prescription smoking cessation medication compared with non-Hispanic white smokers. Similarly, active smokers who were uninsured were less likely to use any prescription smoking cessation medication compared with those privately insured (OR, 0.58; 95% CI, 0.41-0.83). Individuals with ASCVD (OR, 1.35; 95% CI, 1.05-1.75) and COPD (OR, 1.61; 95% CI, 1.27-2.04), compared with those without these conditions, were more likely to use prescription smoking cessation medication.

**Figure 3.**
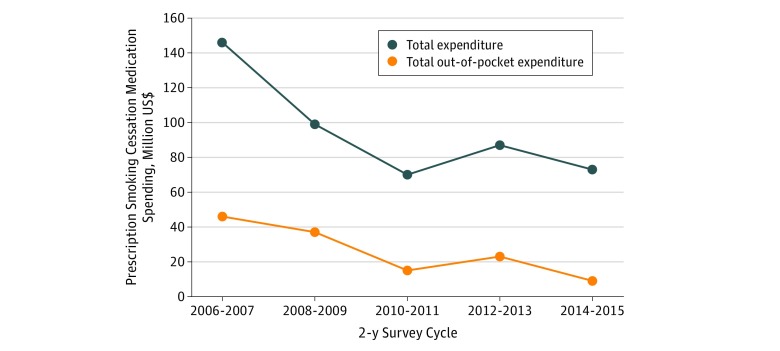
National Trends in Total and Out-of-pocket Expenditures on Prescription Smoking Cessation Medications Among US Adult Smokers, 2006-2015 Data are from the Medical Expenditure Panel Survey.

The annual total expenditures associated with prescription smoking cessation medication use decreased from $146 million in 2006 to 2007 to $70 million in 2010 to 2011, and then spending slightly increased to $87 million in 2012 to 2014 before decreasing again to $73 million in 2014 to 2015 (Figure 3). A similar trend was noted for out-of-pocket expenditures, which decreased from $46 million in 2006 to 2007 to $15 million in 2010 to 2011, and then spending increased to $23 million in 2012 to 2014 before decreasing again to $9 million in 2014 to 2015.

## Discussion

This analysis using nationally representative, population-based samples of current adult smokers over a 10-year period (2006 to 2015) reports several observations. First, the proportion of smokers who reported receiving physician advice to quit smoking at any time within the past year increased between 2006 and 2015, while the uptake of prescription smoking cessation medications decreased, with a corresponding reduction in expenditures associated with prescription use. Second, physician advice to quit smoking was low among certain subgroups, most notably among younger adults; men; Hispanic smokers; uninsured individuals; those living below the FPL; those without a history of smoking-related illness (ie, ASCVD and COPD); and those living in the Midwest, South, or West census regions. Similarly, the uptake of prescription smoking cessation medications was low during the entire 10-year study period and among men, racial/ethnic minority groups, uninsured individuals, and those with no history of a smoking-related illness.

According to a recent report by the Centers for Disease Control and Prevention, cigarette smoking prevalence is at an all-time low.^6^ However, prevalence of smoking remains high among certain subgroups, including young adults, men, racial/ethnic minority groups, those living below the FPL, and those living in the Midwest or South.^6^ The lower rates of physician advice to quit smoking and underuse of prescription smoking cessation medications may be associated with the higher prevalence of cigarette smoking among these subgroups.

Our findings are consistent with those in previous research using national surveys to estimate cessation behaviors among adult smokers in the United States. In a previous analysis from the National Health Interview Surveys, the Centers for Disease Control and Prevention found that self-reported receipt of physician advice to quit smoking increased from about 53% in 2000 to 57% in 2015, but with lower rates among younger adults, racial/ethnic minority groups, and uninsured individuals.^19^ In contrast to the annual MEPS, the National Health Interview Surveys for the supplemental cancer-control questionnaire, which contains the smoking variables used for that analysis, was only administered to participants every 5 years (2000, 2005, 2010, and 2015). Thus, our analysis better captures the trends by providing interannual variability data. Another analysis by Jarlenski et al^20^ found that 16% of cigarette smokers ever filled a prescription for a smoking cessation medication in a nationally representative sample of Medicare beneficiaries between 2007 and 2012. However, a substantial limitation to this finding is that it may not be applicable beyond traditional Medicare populations. Our analyses mitigate this concern given that the MEPS data included all noninstitutionalized adults aged 18 years or older.

The upward trend in the delivery of smoking cessation advice within the health care setting is encouraging and may be associated with the downward trend in smoking prevalence to an all-time low as reported by the Centers for Disease Control and Prevention.^6^ In the United States, most adult smokers who quit smoking do so without a cessation aid.^21^ Thus, a clinician’s advice detailing the health advantages of smoking cessation may suffice for certain patients to attempt quitting smoking.

The discrepant decrease in prescription smoking cessation medication during this same period may be attributed to several theories. It is possible that a great proportion of smokers resorted to cheaper cessation aids such as OTC NRT and emerging alternatives such as electronic cigarettes. This phenomenon may be associated with the corresponding decrease in total and out-of-pocket expenditures for prescription smoking cessation medications seen in this analysis. Yue et al^22^ reported that the uptake of OTC NRT skyrocketed from 2010 to 2015 among Medicaid beneficiaries. Similarly, electronic cigarettes increasingly became popular in the United States in this period and are now the most commonly used smoking cessation aid by US adult smokers trying to quit.^21,23^ Emerging evidence suggest that electronic cigarettes in combination with behavioral therapy are more effective than NRT in achieving smoking cessation.^24^ Although the efficacy of electronic cigarettes over Food and Drug Administration–approved prescription medications like varenicline has not yet been reported, the decrease in smoking cessation pharmacotherapy seen in this analysis possibly reflects a shift from all conventional cessation pharmacotherapies toward electronic cigarettes.

The low uptake of prescription smoking cessation pharmacotherapy among racial/ethnic minority groups, including African American and Hispanic smokers, could be associated with the difference in smoking behaviors, lack of knowledge about smoking cessation therapies, and lack of access to these therapies.^19^ Similarly, low delivery of advice to quit and lower uptake of prescription smoking cessation medications among the uninsured may be attributable to decreased health care use and lack of access to prescription smoking cessation medications. Nonetheless, it may well be that groups with observed disparities constitute smokers who are unwilling to quit and may not acknowledge the receipt of clinician advice. Alternatively, these could also represent those whose clinicians, after many attempts, may have given up on providing both cessation counseling and prescription smoking cessation medications.

We did note the statistically significantly greater odds of the delivery of smoking cessation services among smokers who had a diagnosis of ASCVD and COPD (compared with those without such diagnoses). This disparity may reflect a disproportionate targeting of those with existing smoking-related disease for smoking cessation services over those without clinical disease. Recall bias in persons with smoking-related illness could be associated with the higher number of reports of receiving cessation advice, but recall bias is unlikely to be associated with the higher odds of uptake in prescription smoking cessation medications as medication data were corroborated by the prescribing physicians and pharmacies.

This study reveals a disproportionate and inconsistent delivery of smoking cessation services within the health care setting and raises a question as to whether, in addition to the global approach,^25,26,27,28^ a targeted approach aimed at promoting evidence-based smoking cessation services among vulnerable subgroups (eg, younger adults, men, racial/ethnic minority groups, low income individuals, and the uninsured) will further drive down the prevalence of cigarette smoking among US adults. The Healthy People 2020 initiative objectives on tobacco use include reducing cigarette smoking by adults to 12.0% from 20.6%.^29^ Specific aims include increasing tobacco screening in health care settings by 10% and increasing tobacco cessation counseling in health care settings by 10%.^29^ Routine implementation of clinical guidelines, such as the 2018 American College of Cardiology Expert Consensus Decision Pathway on Tobacco Cessation Treatment, may help improve delivery and uptake of smoking cessation services among vulnerable subgroups. In addition, population-level policies, including barrier-free access to evidence-based tobacco cessation treatments by the uninsured and those living in poverty, antitobacco mass media campaigns tailored for people with different sociocultural backgrounds, and understanding of those with a low level of education, may help attain the Healthy People 2020 goals.

### Limitations

These findings must be interpreted in the context of this study’s limitations. First, because the MEPS data included only the noninstitutionalized US adult population, these findings cannot be generalizable to institutionalized populations such as nursing home residents or incarcerated individuals. Second, receipt of physician advice to quit was self-reported and therefore subject to recall bias, especially by those with or without a smoking-related illness. Third, no information was collected for OTC medications in MEPS. This was an important limitation considering the increasing use of electronic cigarettes in the US population.^21^ Fourth, it was unclear how nonresponse rate in MEPS may affect these findings. Fifth, the associations found in this study may be attributed to residual confounding or other factors not considered in our analysis. For example, previous MEPS have found that individuals with mental illness have higher prevalence of low quit rates compared with those without mental illness.^30^ In this large, population-based study, even small differences will likely meet statistical significance but may not represent clinically significant differences. In this regard, we placed emphasis on associations with large effect estimates.

## Conclusions

In nationally representative cross-sectional surveys of US adult smokers from 2006 to 2015, the proportion who reported receiving physician advice to quit smoking increased. This increase in smoking cessation advice within the health care setting may be associated with the decrease in cigarette smoking to an all-time low among adults in the United States. However, despite this progress, disparities in the delivery of smoking cessation services still remain. The lower rates of delivery of physician advice to quit smoking and the lower uptake of proven prescription smoking cessation medications among men, the uninsured, those living below the FPL, racial/ethnic minority groups, younger adults, and those without smoking-related disease may be associated with the higher rates of smoking among these subgroups despite the overall improvements in smoking quit rates. Thus, in addition to a global approach, a more targeted implementation of smoking cessation guidelines appears to be needed as part of the care for vulnerable subgroups.

## Notes

Supplement. **eFigure 1.** Flow Chart of Patient Selection Process, 2006-2015 Medical Expenditure Panel Survey **eFigure 2.** Trends in Weighted Proportions of US Adult Smokers Reporting Receipt of a Doctor’s Advice to Quit, 2006-2015 Medical Expenditure Panel Survey (By Subgroups) **eTable 1.** ICD-9-CM Codes of Diseases and Self-reported Information **eTable 2.** Trends in Receipt of a Doctor’s Advice to Quit Among US Adult Smokers, 2006-2015 Medical Expenditure Panel Survey **eTable 3.** Trends in Prescription Cessation Medication Use Among US Adult Smokers, 2006-2015 Medical Expenditure Panel Survey
