# Supplementary material for: National Trends in Cessation Counseling, Prescription Medication Use, and Associated Costs Among US Adult Cigarette Smokers
Source: JAMA Netw Open. 2019 May 24;2(5):e194585. doi: 10.1001/jamanetworkopen.2019.4585 (PMC6632149; doi:10.1001/jamanetworkopen.2019.4585)
Supplement: Supplement. — eFigure 1. Flow Chart of Patient Selection Process, 2006-2015 Medical Expenditure Panel Survey eFigure 2. Trends in Weighted Proportions of US Adult Smokers Reporting Receipt of a Doctor’s Advice to Quit, 2006-2015 Medical Expenditure Panel Survey (By Subgroups) eTable 1. ICD-9-CM Codes of Diseases and Self-reported Information eTable 2. Trends in Receipt of a Doctor’s Advice to Quit Among US Adult Smokers, 2006-2015 Medical Expenditure Panel Survey eTable 3. Trends in Prescription Cessation Medication Use Among US Adult Smokers, 2006-2015 Medical Expenditure Panel Survey [file jamanetwopen-2-e194585-s001.pdf]

Supplementary Online Content

Tibuakuu M, Okunrintemi V, Jirru E, et al. National trends in cessation counseling, prescription medication use, and associated costs among US adult cigarette smokers. *JAMA Netw Open.* 2019;2(5): e194585. doi:10.1001/jamanetworkopen.2019.4585

eFigure 1. Flow Chart of Patient Selection Process, 2006-2015 Medical Expenditure Panel Survey

eFigure 2. Trends in Weighted Proportions of US Adult Smokers Reporting Receipt of a Doctor's Advice to Quit, 2006-2015 Medical Expenditure Panel Survey (By Subgroups)

eTable 1. ICD-9-CM Codes of Diseases and Self-reported Information

eTable 2. Trends in Receipt of a Doctor's Advice to Quit Among US Adult Smokers, 2006-2015 Medical Expenditure Panel Survey

eTable 3. Trends in Prescription Cessation Medication Use Among US Adult Smokers, 2006-2015 Medical Expenditure Panel Survey

This supplementary material has been provided by the authors to give readers additional information about their work.

©2019 Tibuakuu M et al. *JAMA Network Open.*

eFigure 1. Flow Chart of Patient Selection Process, 2006-2015 Medical Expenditure Panel Survey

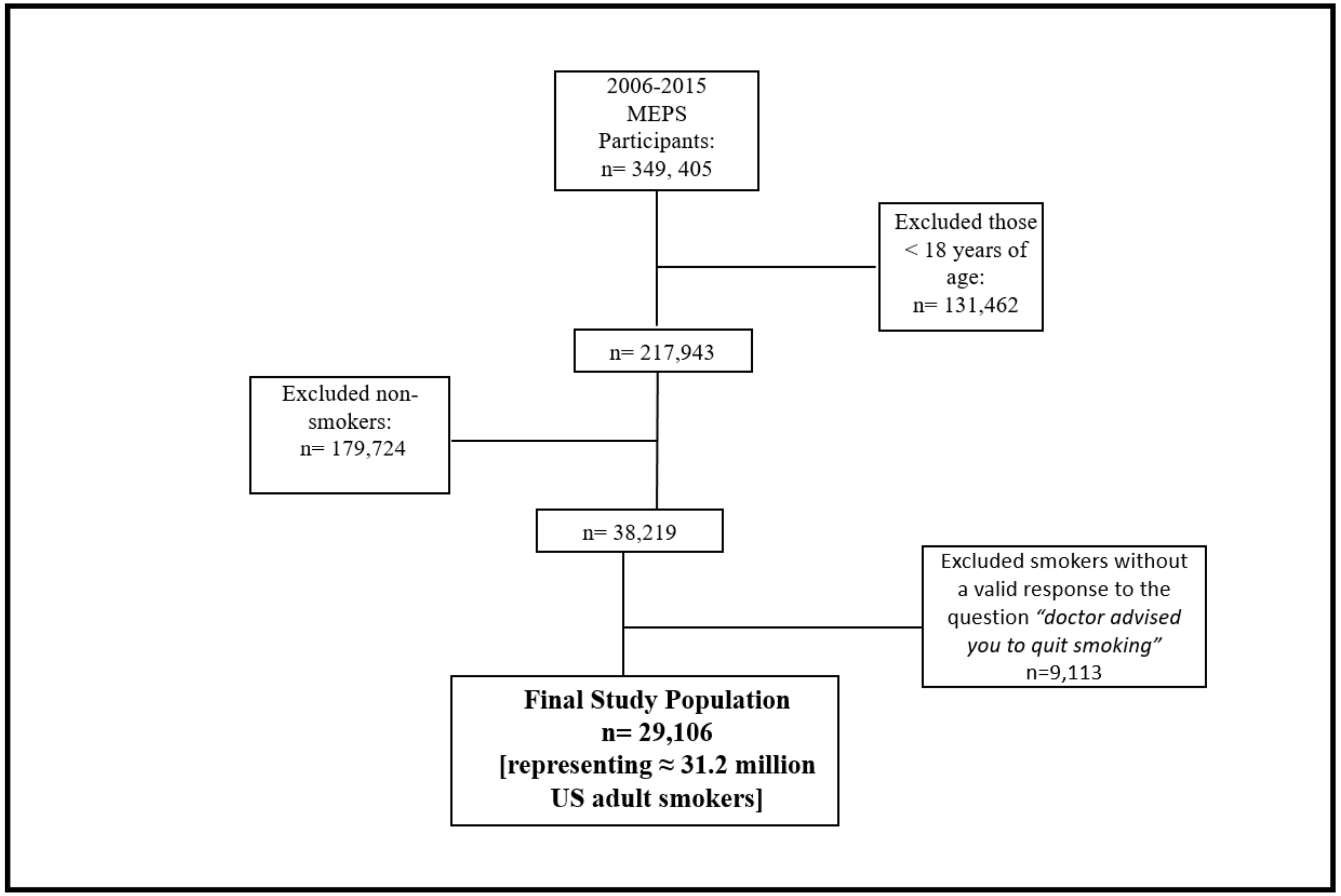

©2019 Tibuakuu M et al. *JAMA Network Open.*

eFigure 2. Trends in Weighted Proportions of US Adult Smokers Reporting Receipt of a Doctor's Advice to Quit, 2006-2015 Medical Expenditure Panel Survey (By Subgroups)

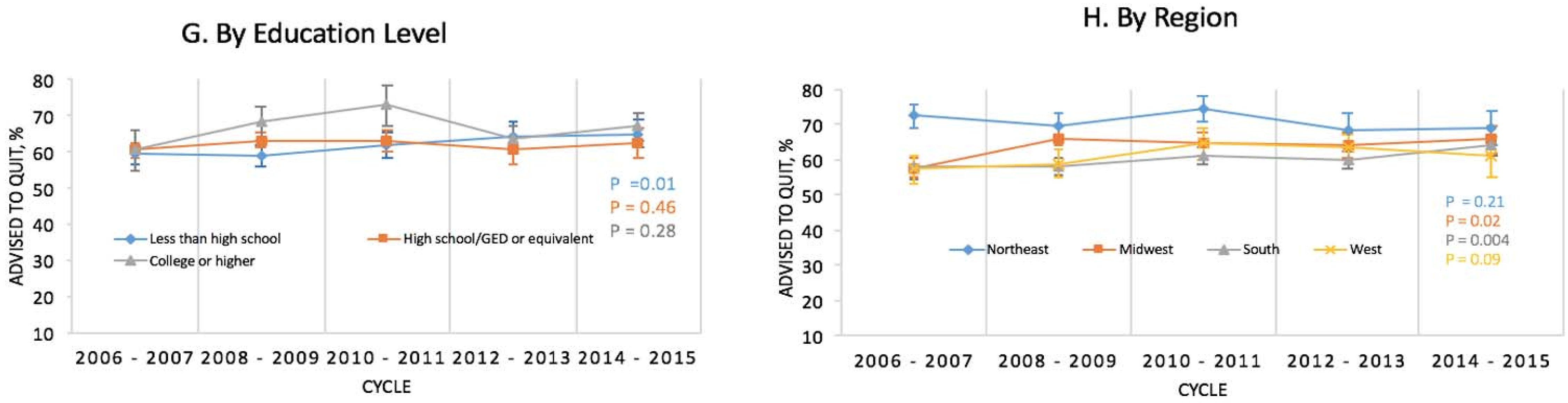

©2019 Tibuakuu M et al. *JAMA Network Open.*

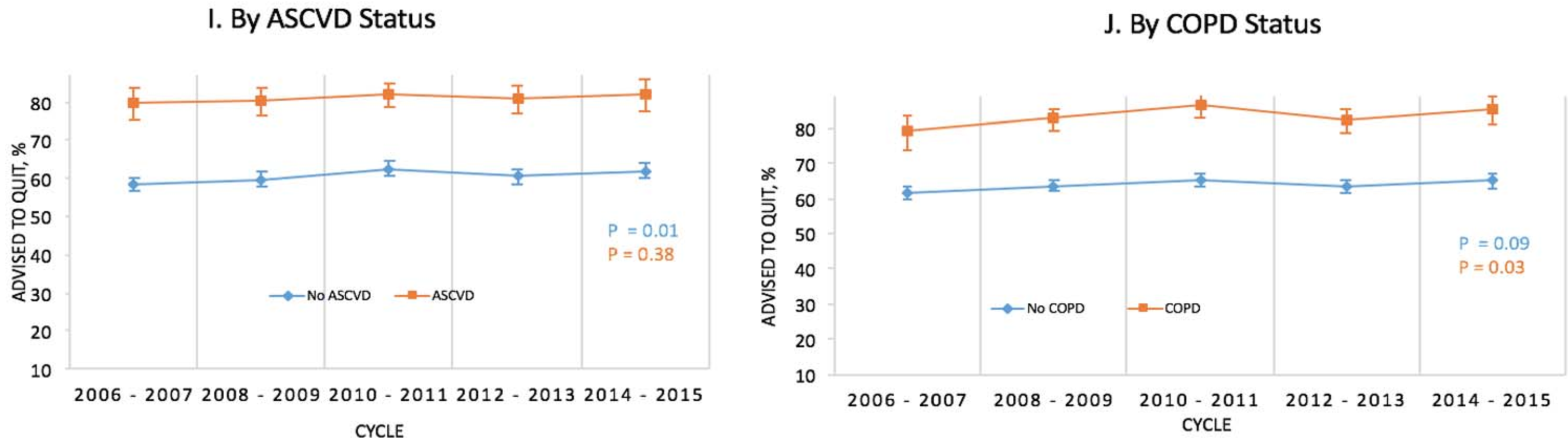

Error bars represent 95% confidence interval; MEPS, Medical Expenditure Panel Survey

©2019 Tibuakuu M et al. *JAMA Network Open.*

eTable 1. ICD-9-CM Codes of Diseases and Self-reported Information

ASCVD and COPD were constructed based on presence of either ICD-9-CM or self-report information	
	**ASCVD**
Condition	*ICD-9-CM Code(s)*
Coronary Artery Disease	410, 413, 414
Stroke	433-437
Peripheral Artery Disease	440, 443
	Self-reported questions for CVD
Coronary heart disease	Asked if the person had ever been diagnosed as having coronary heart disease
Angina	Asked if the person had ever been diagnosed as having angina, or angina pectoris
Myocardial Infarction	Asked if the person had ever been diagnosed as having a heart attack, or myocardial infarction
Stroke	Asked if the person had ever been diagnosed as having had a stroke or transient ischemic attack (TIA or ministroke).
	Self-reported questions for cancer
	Ascertained by asking whether the person had ever been diagnosed as having cancer or a malignancy of any kind.
	**COPD**
Condition	*ICD-9-CM Code(s)*
Bronchitis, not specified	490
Chronic bronchitis	491
Emphysema	492
Bronchiectasis	494
Chronic airway obstruction, not elsewhere classified	496

ICD-9-CM; International Classification of Diseases, 9th Edition, ASCVD; Atherosclerotic Cardiovascular Disease, COPD; Chronic Obstructive Pulmonary Disease.

©2019 Tibuakuu M et al. *JAMA Network Open.*

eTable 2. Trends in Receipt of a Doctor's Advice to Quit Among US Adult Smokers, 2006-2015 Medical Expenditure Panel Survey

	% (95% CI)					p-value for trend
Cycle	2006-2007	2008-2009	2010-2011	2012-2013	2014-2015	
No. of adults	5878	6179	5847	6172	5030	
Weighted sample, million	6.4	6.7	6.3	6.2	5.6	
Overall						
	60. 2 (58.5–62.0)	62.5 (60.8–64.2)	65.0 (63.2–66.6)	63.1 (61.5–64.8)	64.9 (62.8–66.9)	0.001
Age, category, y						
18-39	51.1 (48.2 – 53.9)	53.5 (50.7 – 56.2)	55.2 (52.1 – 58.2)	53.8 (51.0 – 56.6)	55.7 (52.5 – 58.8)	0.05
40-64	65.9 (63.7 – 68.0)	68.1 (65.7 – 70.4)	70.0 (67.8 – 72.2)	67.3 (64.7 – 69.8)	68.8 (66.2 – 71.4)	0.24
65-74	71.2 (65.7 – 76.1)	73.1 (66.6 – 78.8)	81.0 (76.2 – 85.0)	78.2 (72.9 – 82.8)	78.2 (72.2 – 83.2)	0.05
≥ 75	64.6 (55.0 – 72.2)	59.5 (49.6 – 68.7)	69.4 (61.9 – 76.1)	72.1 (61.2 – 80.8)	75.1 (64.1 – 83.6)	0.04
Sex						
Men	56.1 (53.8 – 58.3)	58.1 (55.7 – 60.4)	60.7 (58.3 – 63.0)	59.1 (56.9 – 61.3)	60.0 (57.3 – 62.6)	0.02
Women	65.0 (62.8 – 67.2)	67.5 (65.3 – 69.6)	69.8 (67.4 – 72.1)	67.7 (65.2 – 70.1)	70.3 (67.6 – 72.8)	0.01
Race/ethnicity						
Non-Hispanic white	61.0 (58.9 – 63.0)	64.8 (62.7 – 66.8)	66.2 (64.1 – 68.3)	64.9 (62.7 – 67.0)	66.6 (64.1 – 69.1)	0.003
Non-Hispanic black	61.4 (57.3 – 65.4)	56.3 (52.6 – 60.0)	62.1 (59.2 – 65.0)	60.9 (57.7 – 64.0)	59.7 (56.1 – 63.1)	0.82
Asian	68.4 (56.7 – 78.2)	59.5 (48.2 – 69.8)	66.7 (57.7 – 74.5)	57.1 (50.0 – 64.4)	67.7 (56.5 – 77.2)	0.86
Hispanic	50.0 (44.7 – 55.2)	49.9 (45.6 – 54.3)	57.9 (53.2 – 62.5)	55.2 (50.6 – 59.7)	57.9 (53.5 – 62.2)	0.01
Education						
< High school	59.5 (56.5 – 62.4)	59.0 (56.2 – 61.7)	61.8 (58.2 – 65.4)	64.1 (59.7 – 68.4)	65.0 (61.0 – 68.7)	0.01
High school/GED Equivalent	60.6 (58.5 – 62.7)	63.2 (61.1 – 65.2)	63.2 (60.1 – 66.2)	60.6 (56.8 – 64.3)	62.6 (58.6 – 66.4)	0.46
> some college	60.4 (54.8 – 65.8)	68.1 (63.2 – 72.7)	73.0 (67.2 – 78.2)	63.6 (60.0 – 67.0)	67.3 (63.3 – 71.0)	0.28
Health insurance status						
Private	61.3 (59.0 – 63.6)	66.0 (63.6 – 68.3)	67.2 (64.8 – 69.6)	64.4 (61.9 – 66.8)	65.5 (62.4 – 68.5)	0.09

©2019 Tibuakuu M et al. *JAMA Network Open.*

Public (Medicare/Medicaid)	68.3 (65.1 – 71.4)	67.7 (64.4 – 70.9)	72.2 (69.6 – 74.6)	73.6 (70.7 – 76.3)	70.0 (66.9 – 72.9)	0.13
Uninsured	46.3 (42.7 – 49.8)	44.1 (40.7 – 47.6)	48.6 (45.0 – 52.2)	43.4 (39.6 – 47.4)	45.4 (40.3 – 50.7)	0.71
Family income level						
High income	63.4 (59.9 – 66.8)	67.6 (64.4 – 70.7)	71.0 (67.9 – 73.9)	66.7 (63.0 – 70.3)	67.6 (63.1 – 71.9)	0.15
Middle income	59.1 (56.3 – 61.8)	62.7 (60.0 – 65.4)	66.0 (62.8 – 69.1)	60.6 (57.9 – 63.3)	63.0 (59.2 – 66.1)	0.27
Low income	55.7 (51.5 – 59.8)	56.1 (52.4 – 59.8)	59.5 (55.7 – 63.1)	62.4 (57.8 – 66.8)	65.0 (60.6 – 69.2)	<0.001
Poor	60.3 (56.9 – 63.6)	60.2 (57.7 – 62.6)	61.2 (58.4 – 63.9)	63.1 (60.5 – 65.5)	64.6 (61.1 – 68.0)	0.03
Census Region						
Northeast	72.7 (69.2 – 75.9)	69.7 (65.6 – 73.5)	74.6 (70.7 – 78.1)	68.6 (63.8 – 73.1)	69.1 (64.2 – 73.6)	0.21
Midwest	57.7 (54.9 – 60.5)	66.1 (62.4 – 69.5)	64.5 (61.0 – 67.8)	64.0 (60.2 – 67.6)	65.7 (61.7 – 69.5)	0.02
South	57.8 (54.5 – 61.0)	58.2 (55.9 – 60.4)	61.3 (58.7 – 63.8)	60.0 (57.7 – 62.2)	63.9 (61.1 – 66.5)	0.004
West	57.5 (53.4 – 61.4)	58.9 (54.9 – 62.7)	64.7 (60.1 – 69.0)	63.7 (59.7 – 67.4)	61.2 (55.3 – 66.7)	0.09
Comorbid condition						
No ASCVD	58.3 (56.6 – 60.1)	59.8 (57.9 – 61.6)	62.6 (60.7 – 64.5)	60.5 (58.7 – 62.3)	62.0 (59.9 – 64.1)	0.01
ASCVD	79.9 (75.5 – 83.8)	80.2 (76.2 – 83.7)	82.1 (78.7 – 85.1)	81.0 (77.1 – 84.3)	82.3 (77.8 – 86.1)	0.38
No COPD	61.7 (59.7 – 63.6)	63.3 (62.4 – 65.3)	65.2 (63.2 – 67.1)	63.6 (61.7 – 65.4)	65.2 (63.1 – 67.3)	0.03
COPD	79.1 (74.0 – 83.4)	82.7 (79.0 – 85.6)	86.8 (83.1 – 89.8)	82.3 (78.7 – 85.4)	85.5 (81.3 – 88.8)	0.09

Abbreviations: MEPS, Medical Expenditure Panel Survey; SD, standard deviation; ASCVD, Atherosclerotic Cardiovascular Disease; COPD, Chronic Obstructive Pulmonary Disease.

©2019 Tibuakuu M et al. *JAMA Network Open.*

eTable 3. Trends in Prescription Cessation Medication Use Among US Adult Smokers, 2006-2015 Medical Expenditure Panel						
	% (95% CI)					p-value for trend
Cycle	2006-2007	2008-2009	2010-2011	2012-2013	2014-2015	
No. of adults	5878	6179	5847	6172	5030	
Weighted sample, millions	6.4	6.7	6.3	6.2	5.6	
Overall						
	6.0 (5.2–6.9)	5.3 (4.5–6.1)	4.9 (4.1–5.7)	4.6 (4.0–5.3)	5.5 (4.6–6.5)	0.22
Age, category, y						
18-39	4.3 (3.1 – 5.8)	3.7 (2.8 – 4.9)	4.3 (3.1 – 5.9)	3.6 (2.4 – 5.3)	4.8 (3.6 – 6.7)	0.62
40-64	7.7 (6.5 – 9.0)	6.3 (5.2 – 7.8)	5.3 (4.2 – 6.7)	5.5 (4.6 – 6.7)	6.2 (4.9 – 7.8)	0.06
65-74	4.1 (2.2 – 7.5)	5.8 (3.0 – 11.0)	6.1 (4.2 – 8.7)	4.2 (2.4 – 7.3)	5.1 (2.8 – 9.3)	0.98
≥ 75	2.6 (0.7 – 9.3)	2.4 (0.7 – 7.7)	2.0 (0.7 – 5.8)	1.8 (0.3 – 9.9)	3.1 (0.9 – 10.7)	0.96
Sex						
Men	4.8 (3.9 – 6.0)	4.9 (3.8 – 6.3)	3.8 (2.9 – 5.1)	4.6 (3.6 – 5.8)	5.0 (3.9 – 6.4)	0.97
Women	7.1 (5.9 – 8.6)	5.6 (4.6 – 6.8)	5.9 (5.0 – 7.0)	4.6 (3.7 – 5.7)	6.0 (4.8 – 7.5)	0.13
Race/ethnicity						
Non-Hispanic white	7.0 (5.9 – 8.2)	6.1 (5.1 – 7.3)	5.3 (4.4 – 6.4)	5.0 (4.2 – 6.0)	6.1 (5.0 – 7.5)	0.11
Non-Hispanic black	3.1 (2.1 – 4.5)	2.8 (1.8 – 4.4)	3.4 (2.4 – 4.7)	2.8 (1.8 – 4.4)	3.0 (2.1 – 4.4)	0.93
Asian	4.0 (1.1 – 13.3)	***	***	2.5 (0.7 – 7.8)	***	0.18
Hispanic	1.7 (0.9 – 3.3)	2.5 (1.3 – 4.7)	3.6 (2.1 – 6.0)	4.5 (2.3 – 8.8)	4.0 (2.4 – 6.4)	0.02
Education						
< High school	5.2 (3.9 – 6.9)	4.0 (2.8 – 5.7)	4.6 (3.0 – 7.1)	5.1 (3.4 – 7.7)	4.4 (2.8 – 6.7)	0.75
High school/GED Equivalent	5.8 (4.8 – 6.9)	5.6 (4.5 – 6.8)	4.5 (3.2 – 6.3)	3.7 (2.4 – 5.6)	5.2 (3.4 – 7.8)	0.20

©2019 Tibuakuu M et al. *JAMA Network Open.*

> some college	9.0 (6.5 – 12.3)	6.8 (4.4 – 10.3)	8.7 (5.4 – 13.9)	5.1 (3.6 – 7.1)	5.6 (4.0 – 7.6)	0.02
Health insurance status						
Private	6.6 (5.5 – 8.1)	5.9 (4.7 – 7.4)	5.3 (4.1 – 6.8)	5.2 (4.2 – 6.4)	6.0 (4.8 – 7.6)	0.30
Public (Medicare/Medicaid)	5.6 (4.2 – 7.3)	5.6 (4.1 – 7.5)	5.6 (4.4 – 7.1)	4.7 (3.8 – 5.9)	5.6 (4.2 – 7.3)	0.80
Uninsured	4.7 (2. 9 – 7.6)	2.4 (1.6 – 3.4)	1.3 (0.7 – 2.4)	2.5 (1.4 – 4.4)	2.5 (1.1 – 5.2)	0.08
Family income level						
High income	7.1 (5.6 – 9.0)	6.6 (5.1 – 8.5)	5.4 (3.8 – 7.5)	4.7 (3.3 – 6.8)	5.2 (3.5 – 7.6)	0.03
Middle income	5.1 (3.9 – 6.7)	5.8 (4.6 – 7.2)	4.2 (3.1 – 5.6)	5.8 (4.4 – 7.6)	6.4 (4.7 – 8.7)	0.38
Low income	6.9 (5.0 – 9.6)	3.8 (2.6 – 5.5)	3.6 (2.2 – 6.0)	3.5 (2.3 – 5.4)	4.2 (2.9 – 5.9)	0.05
Poor	5.0 (3.8 – 6.6)	3.9 (3.0 – 5.2)	6.0 (4.7 – 7.6)	3.9 (3.0 – 5.1)	5.6 (4.1 – 7.6)	0.67
Census Region						
Northeast	6.1 (4.1 – 9.0)	5.6 (3.6 – 8.7)	5.6 (3.8 – 8.4)	5.0 (3.7 – 6.7)	5.5 (3.4 – 8.7)	0.63
Midwest	7.1 (5.6 – 8.9)	5.9 (4.3 – 8.1)	4.6 (3.5 – 6.2)	5.4 (4.1 – 7.0)	7.2 (5.5 – 9.5)	0.82
South	5.7 (4.5 – 7.3)	3.5 (2.7 – 4.7)	4.5 (3.4 – 5.9)	4.2 (3.2 – 5.4)	4.9 (3.7 – 6.5)	0.60
West	4.7 (3.2 – 7.0)	7.6 (5.8 – 10.0)	5.6 (3.8 – 8.2)	3.8 (2.4 – 6.0)	4.1 (2.5 – 6.5)	0.17
Comorbid condition						
No ASCVD	5.6 (4.7 – 6.6)	4.8 (4.0 – 5.7)	4.9 (4.0 – 5.8)	4.1 (3.5 – 5.0)	5.3 (4.5 – 6.3)	0.35
ASCVD	9.3 (6.6 – 12.9)	7.6 (5.6 – 10.2)	5.0 (3.6 – 7.1)	7.1 (4.8 – 10.4)	6.4 (4.3 – 9.5)	0.20
No COPD	5.5 (4.6 – 6.5)	4.6 (3.9 – 5.5)	4.4 (3.4 – 5.3)	4.1 (3.4 – 4.9)	5.0 (4.1 – 5.9)	0.51
COPD	10.3 (7.6 – 13.9)	8.2 (6.0 – 11.3)	8.1 (5.8 – 11.2)	7.6 (5.4 – 10.7)	8.6 (6.0 – 12.4)	0.25

Abbreviations: MEPS, Medical Expenditure Panel Survey; SD, standard deviation; ASCVD, Atherosclerotic Cardiovascular Disease; COPD, Chronic Obstructive Pulmonary Disease.

©2019 Tibuakuu M et al. *JAMA Network Open.*
